# Commentary on: Quality of Life Evaluation of Patients Undergoing Lumbar Discectomy Using Short Form 36

**DOI:** 10.5812/aapm.3762

**Published:** 2012-04-01

**Authors:** Chan Hong Park, Sang Ho Lee

**Affiliations:** 1 Department of Anesthesiology and Pain Medicine, Daegu Wooridul Spine Hospital, Daegu, South Korea; 2 Department of Neurosurgery, Wooridul Spine Hospital, Seoul, Korea

**Keywords:** Discectomy, Quality of Life, Low Back Pain

Dear Editor,

I have recently reviewed the interesting article by Farzanegan et al. (1), published in the 2011 issue of the Anesthesiology and Pain Medicine: “Quality of life (QOL) evaluation of patients undergoing lumbar discectomy using Short Form 36”. Lumbar discectomy improved both the physical and mental health subscales in patients’ QOL with chronic disc herniation. The mean improvement in physical health scores was significantly higher in the female patients, than in the male subjects. However, there are no statistically significant differences in the improvement in mental health scores between the two sexes, or their education levels and body mass index’ (BMI). 

Lumbar radiculopathy is a disease, and lumbar discectomy is the most common surgical procedure for patients who are experiencing back and leg pain as a result of herniated discs. It is well-known that surgical procedures such as; percutaneous endoscopic discectomy, lumbar microdiscectomy, or spinal fusion, improve the patients’ QOL after 6 months. Also QOL improves more quickly in surgically treated patients, then in those treated conservatively. The results of this article are consistent with previous studies.

Depression plays a role in pain; it is a major contributor to how pain is processed and coped with, affecting QOL and disability after back surgery. Patients with more depressive symptoms preoperatively, were less likely to achieve clinically significant improvements in disability and QOL following lumbar discectomy (2). Hence, patients with depressive symptoms may benefit from preoperative psychological treatment, with the aim of improving their outcomes. Patients with psychological comorbidities often experience long-term disability from low-back pain. 

In the present study, the Short Form 36 (SF-36) questionnaire was used to assess QOL after lumbar surgery. The SF-36 consists of eight sections, which are; vitality, physical functioning, bodily pain, general health perceptions, physical role functioning, emotional role functioning, social functioning, and mental health. Despite the fact that the SF-36 has a number of advantages for the evaluation of QOL, there is still a need for greater precision in measuring QOL. In addition, this article lacks an interpretation of why educational levels and BMI do not affect the SF-36. In chronic low back pain patients, pain severity is more strongly related to being overweight and psychological factors than to pathophysiological changes (3).

Although, this study has a few demerits, Farzanegan et al. have provided evidence that lumbar discectomy is a safe and effective technique to improve both the physical and mental health subscales of the QOL in patients with chronic disc herniation.
